# Extraction of Diterpene-Phytochemicals in Raw and Roasted Coffee Beans and Beverage Preparations and Their Relationship

**DOI:** 10.3390/plants12081580

**Published:** 2023-04-07

**Authors:** Fábio Junior Moreira Novaes, Maria Alice Esteves da Silva, Diana Cardoso Silva, Francisco Radler de Aquino Neto, Claudia Moraes Rezende

**Affiliations:** 1 Chemistry Department, Federal University of Viçosa, Peter Henry Rolfs Avenue, Viçosa 36570-900, MG, Brazil; 2 Aroma Analysis Laboratory, Chemistry Institute, Federal University of Rio de Janeiro, Avenida Athos da Silveira Ramos, 149, Bloco A, Sala 626A, Rio de Janeiro 21941-895, RJ, Brazil; 3 Laboratory for the Support of Technological Development (LADETEC), Chemistry Institute, Federal University of Rio de Janeiro, Avenida Horácio Macedo, 1281, Polo de Química, Bloco C, Rio de Janeiro 21941-598, RJ, Brazil

**Keywords:** *Coffea arabica*, phytochemistry, bioactive compounds, GC-MS, commercial applications

## Abstract

Cafestol and kahweol are expressive furane-diterpenoids from the lipid fraction of coffee beans with relevant pharmacological properties for human health. Due to their thermolability, they suffer degradation during roasting, whose products are poorly studied regarding their identity and content in the roasted coffee beans and beverages. This article describes the extraction of these diterpenes, from the raw bean to coffee beverages, identifying them and understanding the kinetics of formation and degradation in roasting (light, medium and dark roasts) as the extraction rate for different beverages of coffee (filtered, Moka, French press, Turkish and boiled). Sixteen compounds were identified as degradation products, ten derived from kahweol and six from cafestol, produced by oxidation and inter and intramolecular elimination reactions, with the roasting degree (relationship between time and temperature) being the main factor for thermodegradation and the way of preparing the beverage responsible for the content of these substances in them.

## 1. Introduction

The effects of coffee on human health have always intrigued the scientific community. Coffee beans are a chemically complex matrix of over 2000 substances, which yield hundreds (or thousands) more after roasting [1,2]. It is estimated that more than 1000 new volatile organic compounds are produced and released by the roasting process, according to the number of peaks of substances detected in the output of a coffee roaster [2,3]—this value does not include new substances that do not vaporize and remain in the roasted bean.

In the last two decades, the study on the phytochemistry of coffee has also been directed towards the lipid fraction of the bean, known to be little altered during roasting and extracted for coffee beverages during their preparation [4,5,6,7]. This represents up to 17% *w*/*w* of the percentage chemical composition of green beans (raw; not roasted), known for the presence of free and esterified fatty acids to glycerol (mono, di and triacylglycerols; 75–96%), alcohols and fatty ester diterpenes (up to 0.4 and 18.5%, respectively), aliphatic hydrocarbons (0.7–2.2%), sterols (0.5–2.2%), tocopherols (0.002–0.05%), phosphatides (0.3%) and serotonin amides (≤1%) as the main constituents [2,8,9]. Pharmacological and dermo-cosmetic properties are described for these classes of compounds. 

A broad bioactive profile of these diterpenes is characterized by antioxidant, anti-inflammatory, anti-adipogenic, anti-diabetic and anti-carcinogenic activity [10,11,12,13], which leads coffee oil to be seen differently in terms of its potential, already demonstrated in studies of photoprotective effect, healing, moisturizing and hypoglycemic properties [14,15,16,17]. 

**Figure 1:**
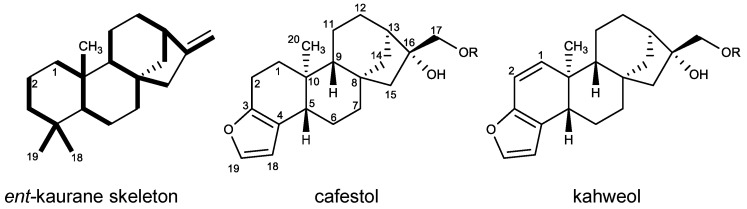
Chemical structure of green Arabica coffee diterpenes (Free form: R = H; Esterified form: R = fatty acid acyl chain with n-C_14_–C_24_).

The diterpene esters present in Arabica coffee (*Coffea arabica* L., Rubiaceae) are formed by aliphatic fatty acids with different chain lengths and degrees of unsaturation (C_14:0_, C_16:0_, C_17:0_, C_18:0_, C_18:1_, C_18:2_, C_18:3_, C_20:0_, C_20:1_, C_21:0_, C_22:0_, C_23:0_ and C_24:0_) [18], conjugated to cafestol and kahweol (C&K)—two furane-diterpenes with an *ent*-kaurane pentacyclic skeleton, distinguishable only by unsaturation between carbons 1 and 2 found in kahweol and absent in cafestol [19] (Figure 1). Even after adding the free (di-alcoholic) and esterified form, C&K correspond to less than 3.5% *w*/*w* of the chemical composition of the beans, where they are stored together with other compounds as lipid corpuscles in the green bean cavities [8,18,20].

The esterified form of C&K diterpenes is the most abundant in coffee beans (99.6%), and it is an analytical challenge due to the structural similarity of the 26 C&K esters. Therefore, research has been concentrated on the free structures after hydrolysis of C&K esters, simplifying their determination in coffee matrices and enhancing their quantity for various studies, either for their quantification in raw and roasted beans [21,22,23,24] or beverages [25,26,27,28,29,30] as well as for biological activity assays [10,31,32,33,34].

Cafestol and kahweol alcohols and esters are partially degraded during roasting, depending on the temperature and exposure time employed [35,36]. Some C&K degradation products are described in the literature, which preserve the *ent*-kaurane pentacyclic skeleton (Figure 1). Along with diterpenes, these derivatives can be extracted during the preparation of beverages, whose concentration depends on the proportions between ingredients, method and preparation time [37,38]. 

In the boiled coffee beverage, it is possible to find up to 1.766 mg L^−1^ of diterpene esters—950 mg L^−1^ of the respective C&K alcohols—while in beverages prepared with filter paper, these phytochemicals are highly retained in the coffee grounds, allowing the percolation of only 8.1 mg L^−1^ of esters and 4.4 mg L^−1^ of diterpene alcohols [27,37,38]. However, little is known about the degradation products of these molecules and their respective formation and degradation kinetics along the roasting profiles, identity, levels and their effects on human health.

To identify and quantify the free C&K diterpenes of Arabica coffee and their degradation products formed in the final processes of the commodity, we determine the mass balance of these compounds from green beans and investigate their degradation kinetics throughout the process of roasting (light, medium and dark) and chemical characterization by gas chromatography coupled to mass spectrometry (GC-MS), followed by the extraction and analysis of hot coffee beverages (boiled, filtered, Moka, French press and Turkish).

## 2. Results and Discussion

### 2.1. Diterpene Content in Green and Roasted Coffee Beans

**Figure 2:**
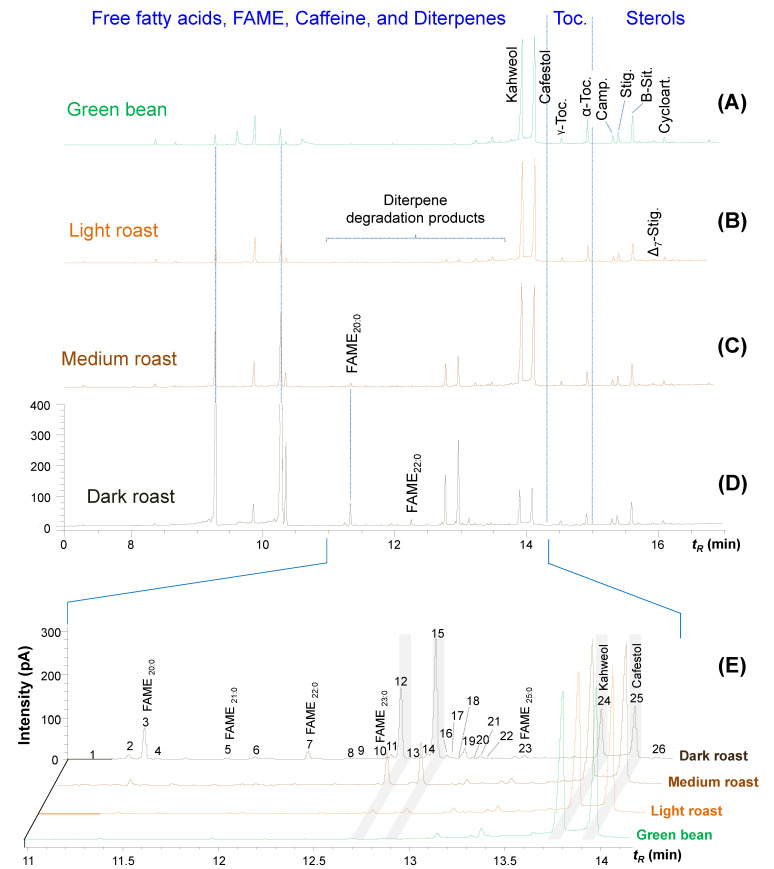
GC-FID chromatograms of TBME extracts from metabolized Arabica coffee oil obtained from green (**A**) and roasted beans: (**B**) light roast, (**C**) medium roast and (**D**) dark roast. (**E**) Superimposition of A–D chromatograms with the expansion of the region between 11.0 to 14.3 min (numbered peaks identified in Table 2).

To determine the mass balance of diterpenes in coffee between green and roasted beans, the methanolysis reaction of the oil was performed followed by the extraction of the saponifiable fraction and GC-MS analysis. Figure 2 presents the GC-FID chromatograms of the methanolized lipid fraction of green and roasted beans, allowing for a correlation between the retention times (*t_R_*) of each peak and their abundance. Caffeine, fatty acids, fatty acid methyl esters (FAME), diterpenes, tocopherols and steroids were observed (Figure 2). 

The increase in lipid component content results from the roasting of coffee beans [8,36,39] is due to the release of the remaining oil, once trapped in the cavities of the green bean, since the roasting process causes hydrolysis and rupture of the cell walls formed by polysaccharides after the bean bursts—a phenomenon known in the coffee industry as popping or cracking—due to the expansion and release of water vapor, CO_2_ and newly produced volatile organic compounds, thereby, opening the way for the exclusion of oil [3,40,41] and the elevation of its content in the obtained extracts. 

The more intense the roasting, the greater the fragility of the bean walls due to the hydrolysis and thermo-oxidation of the polysaccharide structures that make up the walls of the beans, followed by the release of the intracellular content to the surface of the roasted bean. On the other hand, the roasting process promotes pyrolysis reactions. These reactions explain the degradation of diterpenes observed in roasted bean extracts and intensify with increasing temperature and roasting time: light roast: 230 °C and 12 min < medium roast 240 °C and 14 min < dark roast: 250 °C and 17 min (item 4.2).

The greater fragility of roasted beans would make them a better matrix for oil extraction by pressing or solid–liquid extraction under optimized roasting conditions to control their chemical composition. Roasted coffee oil is useful for applications in the food industry as a flavor (for candies, ice creams, chocolates, etc.) and in the cosmetics industry due to its antioxidant, moisturizing, healing and protection against UV-B rays [14,15,42,43,44].

In addition to the increase in peak intensity, Figure 2A–D shows changes in the composition of the extracts due to the presence of FAMEs (C_20:0_, C_21:0_, C_22:0_, C_23:0_ and C_25:0_), α-tocopherol, steroids and other compounds not previously observed and, mainly, the thermodegradation products of C&K diterpenes. The other lipid components of coffee are more thermally stable than these diterpenes and, therefore, little changed. Although the products of lipid thermo-oxidation are known (namely, carboxylic acids, alcohols, aldehydes, ketones, esters and hydrocarbons), all of them with varying chain lengths (depending on the triacylglycerols present), saturated and unsaturated and free and epoxidized, some of which have already been found in the smoke released by coffee roasters after popping the bean, were not observed in the present work [3,40].

The amount of C&K in the green Arabica coffee bean was 0.8% *w*/*w* (797 mg 100 g^−1^ of coffee beans) (Table 1), which is consistent with the literature data, which presents a range between 0.7 and 1.3% for Brazilian Arabica beans [24,45,46] and up to 3.5% for those from other producing countries [8]. As expected, there are no diterpene degradation products in the green coffee bean; if observed, they would be a result of the GC injection system due to the thermolability of these compounds [24,47]. From light roasting, the appearance of degradation products of C&K diterpenes is observed, which increases proportionally as the intensity of roasting (Figure 2 and Table 1).

**Table 2:** Identified coffee components in the TBME extract of green and roasted beans by GC–MS based on matching the molecular mass MS NIST library (Match and Relative Match) with experimental retention index (*I_Exp_*). The X indicates the presence of the compound in the extract.

*t_R_*	Name	Molecular Mass	Match/ R. Match	*I_Exp_*	Present in Coffee Bean			
					Green	Light	Medium	Dark
9.188	Palmitic acid, methyl ester	270	947/947	1971	X	X	X	X
9.405	Ferulic acid, methyl ester	208	955/955	2010			X	X
9.524	Palmitic acid	256	948/953	2032	X	X	X	X
9.767	Caffeine	194	959/959	2077	X	X	X	X
10.015	Linoleic acid, methyl ester	294	909/911	2123	X	X	X	X
10.227	Stearic acid, methyl ester	298	892/898	2164	X	X	X	X
10.528	Linoleic acid	280	904/936	2222	X	X	X	X
11.025	Anhydrous cafestol derivate (#1)	282	-	2322				X
11.116	Eicosenoic acid, methyl ester (#2)	324	957/920	2356				X
11.187	Arachidic acid, methyl ester (#3)	326	779/789	2341	X	X	X	X
11.347	Anhydrous cafestol derivate (#4)	282	-	2389				X
11.752	Heneicosanoic acid, methyl ester (#5)	340	778/823	2477				X
11.915	Linolein, 2-mono- (#6)	354	753/825	2555				X
12.222	Behenic acid, methyl ester (#7)	354	938/950	2581			X	X
12.486	Kahweal (#8)	296	-	2644		X	X	X
12.553	Dehydro-kahweol derivate (#9)	296	-	2660		X	X	X
12.660	Tricosanoic acid, methyl ester (#10)	368	752/795	2694				X
12.697	Dehydro-kahweol derivate (#11)	296	-	2695		X	X	X
12.759	15,16-Dehydro-kahweol (#12)	296	-	2710		X	X	X
12.855	Dehydro-kahweol derivate (#13)	296	-	2733				X
12.898	Cafestal (#14)	298	-	2744		X	X	X
12.961	15,16-Dehydro-cafestol (#15)	298	-	2759		X	X	X
13.018	Methyl epoxystearate (#16)	312	764/866	2772				X
13.048	Dehydro-kahweol derivate (#17)	296	-	2779				X
13.065	Kahweol derivative (#18)	298	-	2783			X	X
13.122	Dehydro-kahweol derivate (#19)	296	-	2797			X	X
13.170	Cafestol derivative (#20)	300	-	2809		X	X	X
13.206	16-O-isobutyl-kahweol (#21)	370	-	2818				X
13.382	16-O-isobutyl-cafestol (#22)	372	-	2869				X
13.528	Pentacosanoic acid, methyl ester (#23)	396	825/889	2898	X	X	X	X
13.916	Kahweol (#24)	314	-	2993	X	X	X	X
14.105	Cafestol (#25)	316	-	3044	X	X	X	X
14.239	Seco-kahweol (#26)	314	-	3080				X
14.544	γ-Tocopherol	416	907/914	3161	X	X	X	X
14.894	α-Tocopherol	430	858/917	3258	X	X	X	X
15.345	Campesterol	400	898/912	3383	X	X	X	X
15.428	Stigmasterol	412	902/910	3403	X	X	X	X
15.653	β-Sitosterol	414	954/957	3436	X	X	X	X
15.757	5-Avenasterol	412	788/874	3451	X	X	X	X
15.909	Stigmast-7-en-3-ol (Δ-_7_-Stigmastenol)	414	804/855	3474		X	X	X
15.981	Cycloartenol	426	916/896	3484		X	X	X
16.151	N.I.	-	-	-			X	X

The total diterpene contents (last column of Table 1) have a different behavior since a significant increase is found when compared to beans subjected from green (0.8% *w*/*w*) to light and medium roasting, 1.0 and 1.3% *w*/*w* (1.005 and 1.297 mg 100 g^−1^ of coffee beans), increases of 26% and 63% compared to the green bean, respectively. This is followed by a drastic reduction of the total content of diterpenes in dark roasting, caused by the increase in temperature and roasting time (740 mg 100 g^−1^) (Table 1). 

As already mentioned, it is believed that the cell walls that imprison coffee lipids are partially degraded and allow the release of these compounds along with the oil, which justifies the increase in the total lipid content observed in coffee beans obtained from light and medium roasting compared to green beans. This oil leakage during roasting can occur without changing its chemical composition, including the profile of C&K diterpenes, which should change only when the beans are subjected to more intense roasting. This hypothesis is based on the non-appearance of degradation products at high levels in light and medium roasts associated with the increase in C&K levels. 

Much lower values of C&K in beans subjected to dark roasting come from the continuous pyrolysis of these diterpenes as well as their degradation products since they also may have reduced levels (Figure 2D,E; Table 1). Possibly, these are transformed into smaller molecules that are vaporized by the torrefaction process or degraded to polar compounds that were not extracted by the organic solvent used and, therefore, were not observed in the chromatograms presented in Figure 2. 

In addition, C&K and its degradation products are vaporizable substances and migrate to the gaseous phase when exposed to the roasting temperature, thereby, reducing their levels in the solid matrix (Table 1). Fischer et al. [48] and Czech et al. [49] analysed the evolved gas from a coffee roaster by Photoionization Time-of-Flight Mass Spectrometry, where they found molecular ions of *m/z* 296, 298, 314 and 316 in Arabica coffee beans and 298, 316 and 330 in Robusta coffee beans. The authors correlated these ions with the presence of dehydro-kahweol (M^•+^ = *m/z* 296), dehydro-cafestol (*m/z* 298), kahweol (*m/z* 314), cafestol (*m/z* 316) and 16-*O*-methyl-cafestol (*m/z* 330), respectively.

### 2.2. Identity of C&K Degradation Products

Figure 2E shows the overlapping of the chromatograms with an expansion of the elution region of the C&K diterpenes and their thermal degradation products, eluted between 11 to 14 min. About 26 peaks appear and were partially identified, including 6 that are fatty acid methyl esters (peaks #2, 3, 5, 7, 10 and 23), a monoacylglycerol (#6) and an epoxy derivative (#16), kahweol (#24), cafestol (#25) and 16 derivates of these diterpenes produced from the roasting process (Figure 2E). Chromatographic and molecular identification information about the compounds present in the chromatograms in Figure 2 is presented in Table 2.

The increase in roasting intensity promotes the degradation of C&K diterpenes by forming at least 16 new compounds, 10 derived from kahweol and 6 from cafestol (Figure 2E and Table 2). This number reflects the greater thermal instability of kahweol compared to cafestol, already mentioned by several authors regarding its photo and thermodegradation [21,22,24,45,50,51,52,53]. 

Speer & Kölling-Speer [8] identified C&K derivatives produced during roasting, from which the loss of intramolecular water by dehydration of the tertiary hydroxyl gives rise to the derivatives 15,16-dehydro-kahweol and 15,16-dehydro-cafestol, in addition to the kahweal and cafestal aldehydes, which possibly have an *enol* derivative as an intermediary with a double bond between carbons 16 and 17, forming the reaction intermediates that would be the 16,17-dehydro-diterpenes. In addition to these, the authors identified *seco*-kahweol, *iso*-kahweol and 15,16-dehydro-*iso*-kahweol. Among these last three structures, the first comes from the rupture of the B ring of the *ent*-kaurane pentacyclic skeleton of kahweol. The second is formed by rearrangement of the double bond (final position between carbons 5 and 6) throughout the roast. 

Although the authors found *iso*-kahweol in a roasted bean, it was observed by our research group in green Arabica coffee beans, which therefore suggests that it does not have its origin in the process of roasting but would be an endogenous substance of the bean and also a taxonomic marker among the various existing variations of the Arabica coffee species [24]. As for 15,16-dehydro-*iso*-kahweol, it can be produced during roasting from *iso*-kahweol, such as *iso*-kahweal and other derivatives.

Guerrero et al. [47] mentioned the existence of other dehydro-diterpenes (unsaturation between carbons 13–16 or 11–12) of C&K and ether derivatives (16-*O-iso*-butyl-kahweol and 16-*O-iso*-butyl-cafestol), which were all produced inside the injector (in split and splitless modes) of the gas chromatograph since the authors analysed extracts from green Arabica coffee beans. This observation had already been suggested by other authors who found some of these compounds but did not identify them [50,51,54]. These authors proceeded to analyse C&K by GC after derivatization arising from the silanization reaction of the sample [55,56] or directly by HPLC [20,21,22,25,26,27,28,45,53,54,57,58].

Pacetti et al. [56] investigated the saponifiable composition of roasted 100% Arabica coffee beans and found, in percentage values, the diterpenes kahweol (40–52%), cafestol (26–38%) and some of their degradation products, such as dehydro-kahweol (3.3–5.6%) and dehydro-cafestol (2.8–5.7%), without identifying the position of the double bond, in addition to a diterpene (0.3–0.8%) with a molecular mass equivalent to that of kahweol and 16-*O*-methyl-kahweol (0.2–0.4%). The authors did not discuss the presence of this ether-diterpene, observed for the first time in Arabica coffees.

For decades, 16-*O*-methyl-cafestol (16-OMC) was described as a diterpene unique to green Robusta coffee and, therefore, defined as a marker of adulteration in Arabica coffee beans [55,59,60,61]. However, in an unprecedented way, through the use of low-field ^1^H NMR, Gunning et al. [62] identified 16-OMC in 100% Arabica coffee beans after modifying the sample preparation method to obtain more concentrated extracts from roasted beans.

**Figure 3:**
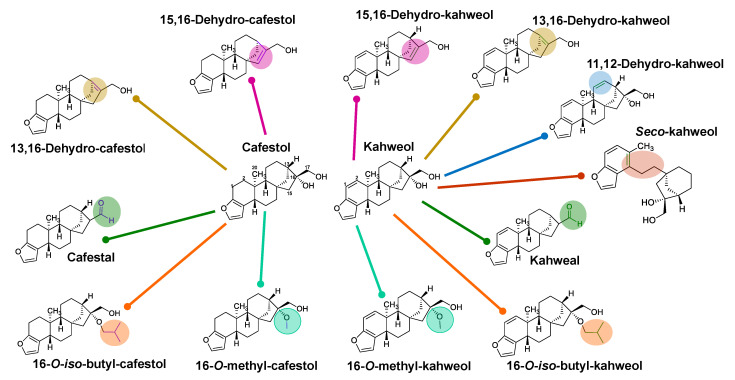
Chemical structure of coffee terpenes—cafestol and kahweol—and their thermal degradation products after [2,8,47,56,62,63].

Figure 3 shows the structures of 12 of the 16 substances mentioned above that would be produced from C&K during roasting. Of all these, only three were isolated using the countercurrent chromatography technique followed by preparative HPLC and had their structures confirmed by ^1^H and ^13^C NMR—namely, the derivatives 16-*O*-methyl-cafestol, 15,16-dehydro-cafestol (#15) and 15,16-dehydro-kahweol (#12) [52].

**Figure 4:**
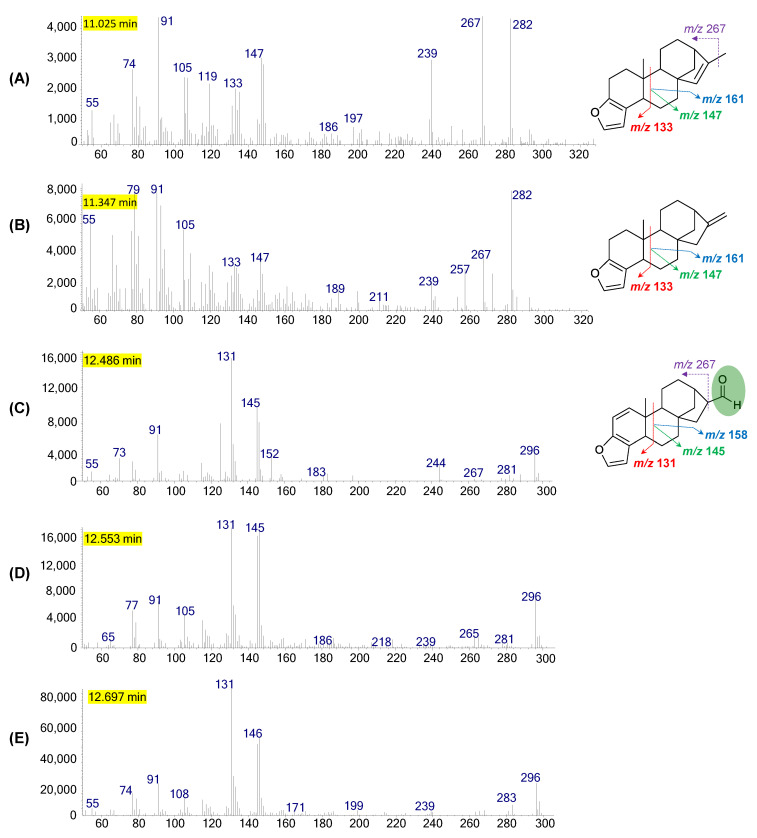

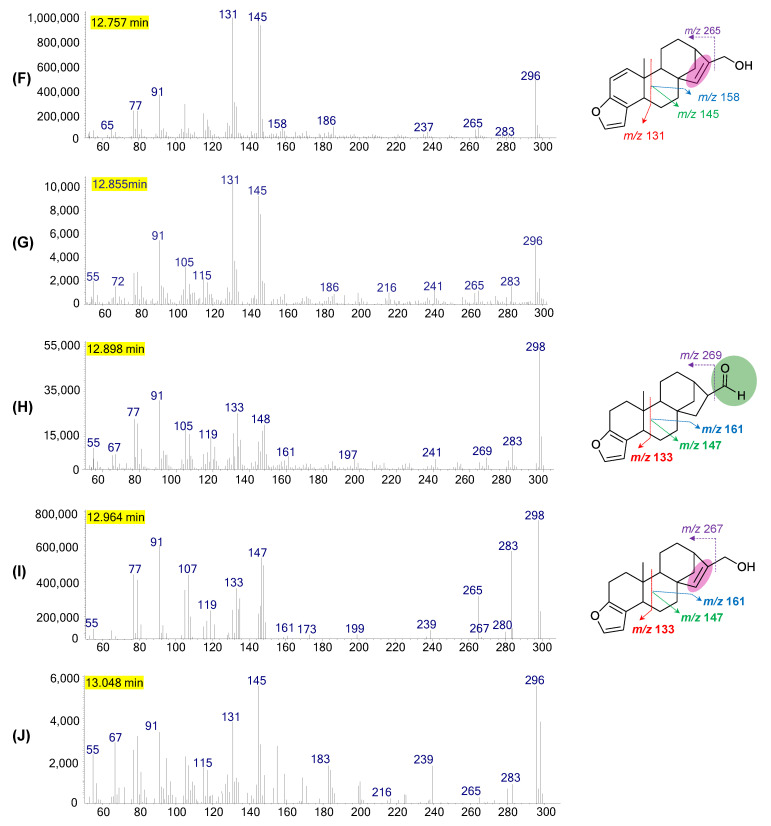

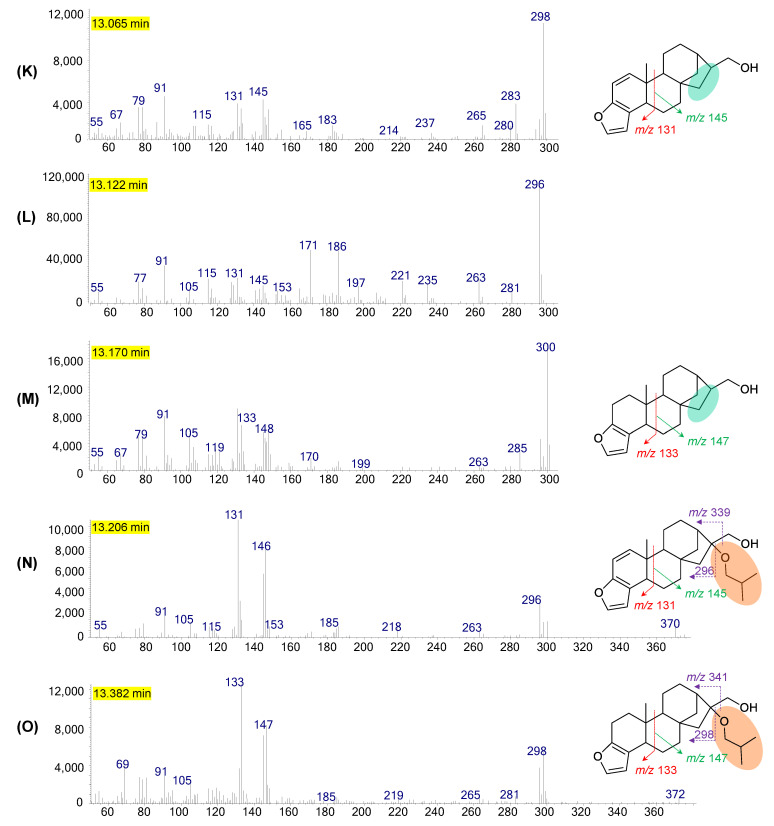

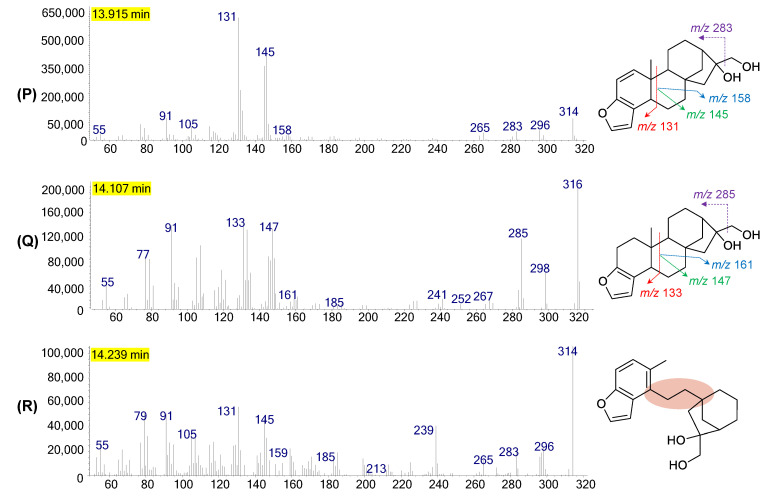
GC-MS mass spectra and chemical structure of coffee diterpenes and their degraded products with theoretical fragmentation pathways proposal.

Fragmentation of the *ent*-kaurane pentacyclic skeleton of coffee diterpenes produced the ions *m/z* 131, 145 and 158 for kahweol derivatives (Figure 4P) and the corresponding ions *m/z* 133, 147 and 161 for cafestol (Figure 4Q), all originating from the same breaks that occur in the B ring. The ions *m/z* 296, 283 and 265 for kahweol (and *m/z* 298, 285 and 267 for cafestol) come from the loss of water due to the elimination of tertiary hydroxyl linked to carbon 16 [M-H_2_O]^+•^, of the methoxy group containing carbon 17 [M-OCH_3_]^+^ and, finally, the one produced by both parts [M-H_2_O-OCH_3_]^+^, respectively. 

These are diagnostic ions for the characterization of C&K diterpenes, which helped to identify their thermal degradation derivatives in the chromatograms obtained from roasted beans (Figure 2). From these data, the presence of eight dehydration derivatives from kahweol (#8–9, 11–13 and 17–19) and only five (#1–2, 14–15 and 20) from cafestol, including the kahweal (#8) and cafestal (#14) aldehydes produced by oxidation of the terminal alcohol function (Table 2). 

All these had the ions related to the breakage of the B ring and as molecular ion [M]^+•^ the characteristic dehydration, namely the ions *m/z* 296 and 298 for dehydro-kahweol and dehydro-cafestol derivates, respectively, followed by the losses (i) of methyl bonded to carbon 10 [M-CH_3_]^+^, (ii) to hydroxyl to carbon 17 [M-OH]^+^ and (iii) to methyl and water [M-CH_3_-H_2_O]^+^, thus, forming ions *m/z* 281, 279 and 263 for dehydro-caveol derivates (Figure 4D–G,J,L) and the corresponding ions *m/z* 283, 281 and 265 for dehydro-cafestol derivates (Figure 4I). 

15,16-Dehydro-diterpenes are considered to be the major degradation products of C&K [8,52] and are proposed as the most abundant peaks in Figure 2 (#12 and #15) and F and I spectra from Figure 4. Cafestal [M]^+•^ *m/z* 298 (Figure 4H), a function isomer of 15,16-dehydro-cafestol (Figure 4I), was distinguished from it by the presence of the ions *m/z* 269 [M-CHO]^+^, referring to the breakage of the alpha bond with the aldehyde function as well as the absence of ions *m/z* 281 and 265. Kahweal is less abundant than cafestal [8], likely due to the greater instability of the molecule produced from kahweol. In general, a fragmentation route equivalent to cafestal is observed for kahweal, namely the ions *m*/*z* 296, 267, 145 and 131, with the absence of ions *m/z* 278 and 263 (Figure 4C).

Two peaks were observed with mass spectra containing the diagnostic B-ring ions for kahweol and cafestol with molecular ions *m/z* 298 and 300 (Figure 2: #18 and #20; Figure 4K,M). The suggested structure refers to the loss of OH (16 Da), indicating intermolecular dehydration—that is, the departure of OH from these diterpenes with acidic hydrogen from the matrix.

Ether derivatives were also observed in the elution region between dehydro-C&K and kahweol, the same ones found by Guerrero et al. (2005), 16-*O*-butyl-kahweol (#21) and 16-*O*-butyl-cafestol (#22). These were also identified by pairs of ions *m/z* 131/145 and 133/147 (due to rupture of the B ring in kahweol and cafestol, respectively), together with the molecular ions *m/z* 372 and 370 [M]^+•^ for 16-*O*-butyl-diterpene derivatives (Figure 4N,O). 16-*O*-methyl-kahweol and 16-*O*-methyl-cafestol were not observed, which have equal molecular ions *m/z* 328 and 330 [M]^+•^, respectively.

*Seco*-kahweol (#26, Figure 2E and Figure 3) was also observed shortly after cafestol. This has the same ions as kahweol and later elution, which is justified by the greater possibility of interaction due to the continuous surface of contact on the stationary phase due to the opening of the B ring, which causes it to have a lower vapor pressure than does a cyclic structure, such as kahweol (Figure 3, #26). Furthermore, the molecular ion (M^•+^ *m/z* 314) of *seco*-kahweol is more intense than that of kahweol, justified by the high stability of the molecule generated by the conjugated aromatic system between the furan ring and the six-membered ring (Figure 4R).

Two other degradation products of cafestol (#1 and #2) were also observed in the elution region of fatty acids and methyl esters, with ions characteristic of the furan group (*m/z* 133, 147 and 161) and apparent molecular ions of *m/z* 282 (Figure 4A,B). These are anhydrous derivatives of cafestol, likely resulting from an intramolecular and an intermolecular elimination, that is, the loss of water and a hydroxyl with a rearrangement for the presence of only one unsaturation, with a probable location between carbons 15–16 (or 13–16) for the first detected molecule and 16–17 for the second. 

The justification for these proposals is due to the more intense ion *m/z* 267 in the first structure due to the sum of the losses of methyl groups linked to carbons 10 and 16 (Figure 4A), while the second structure has only one methyl loss from carbon 10 (Figure 4B). However, for a better structural distinction of these isomers, such as dehydro-kahweol derivatives, the use of computational techniques and the HOMO, LUMO and gap parameters may indicate chemical reactivity and molecular stability to help in the prediction.

These presented results again confirm the thermolability of C&K diterpenes and some structures of their degradation products. Finally, it remains to determine the unequivocal chemical structure of all these molecules, their formation and degradation kinetics within roasting profiles (including those not described in this work) for other coffees of the Arabica and Robusta species, as well as their extraction for the various coffee beverages and the toxicity levels of these molecules, if any.

Molecular and diagnostic fragment masses of all green and roasted coffee diterpenes are provided in the Supplementary Materials (Table S1), identified according to their fragmentation pathway proposals.

### 2.3. Diterpene Content in Coffee Beverages Prepared by Different Coffee Makers

In order to obtain a method for extracting diterpenes and their degradation products in coffee beverages, the improved procedure described by Moeenfard et al. [28] regarding the amount of KOH used by the authors (1.5 g mL^−1^ equivalent to 21 mol L^−1^), which is ten-times higher than the method used as the basis by the authors [26] and twenty-one-times more concentrated than the solution used in this work to obtain diterpenes from coffee beans (item 3.1). In addition, it has been described that diterpenes are unstable to alkaline catalysts as well as the presence of light, high temperatures, molecular oxygen and acid catalysts [21,23,51]. 

Thus, the relationship between stability and extraction efficiency was studied regarding the concentration of the base used in the Turkish coffee beverage, using 0.3, 1.0 and 3.0 g of KOH in the reaction followed by the extraction of the diterpenes present in the beverage. However, no degradation products were observed; however, less extraction efficiency was reached when masses smaller than 3.0 g of KOH were used (Supplementary Materials Table S2). This conclusion was also observed in a previous study by the group for green beans [64], where it was concluded that the saline effect would be responsible for the extraction efficiency when using higher levels of the base, contrary to the literature that pointed to the instability of diterpenes against a strong base, such as KOH [21,23]. 

Thus, we decided to maintain the amount of 3.0 g of KOH for the hydrolysis reaction of the diterpene esters in the beverage. It is believed that the high ionic strength governs the extraction efficiency of free diterpenes from the beverage and that the base does not promote the degradation of C&K [64]. Furthermore, the presence of water makes the catalyst less reactive compared to methanol or ethanol medium—alcohols commonly used for alkaline hydrolysis reactions of esters of fatty acids and triacylglycerols.

**Figure 5:**
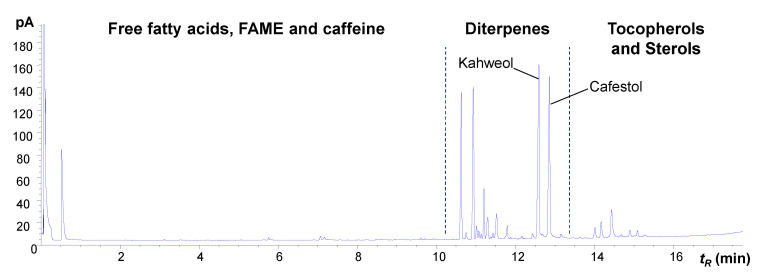
CG-FID chromatogram of the coffee beverage extract from the French Press coffee maker. Beverage obtained from beans subjected to dark roasting.

**Figure 6:**
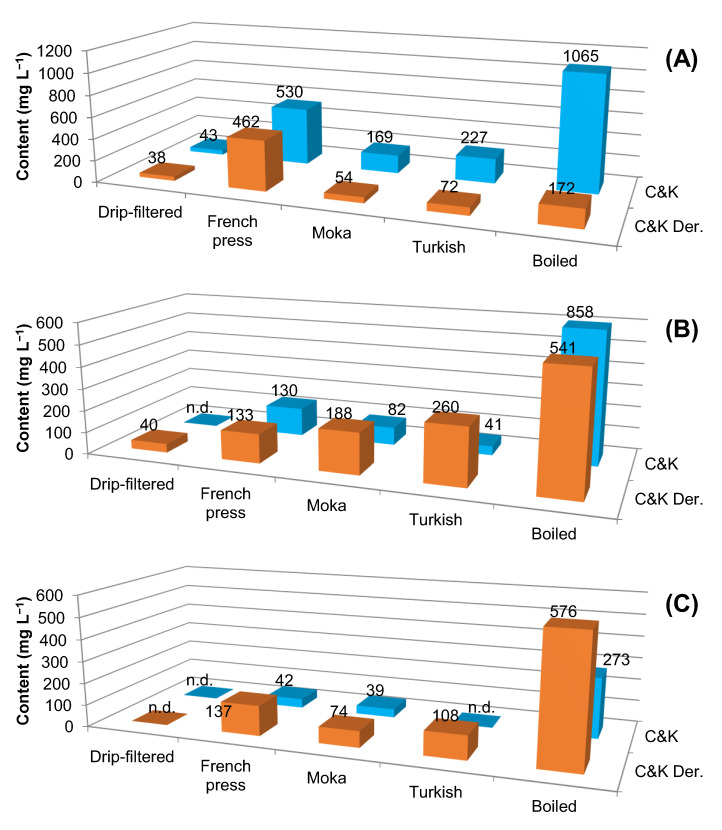
Variation in the levels of diterpenes cafestol and kahweol (C&K) and their degradation derivatives (C&K-Der.) in different coffee beverages, all derived from beans subjected to roasting levels (**A**) light, (**B**) medium and (**C**) dark.

The use of the Moeenfard et al. [28] method was then adopted for the extraction of C&K diterpenes and their degradation products in different hot coffee beverages (filtered, boiled, Moka, French press and Turkish), which were all prepared using roasted coffee beans at the three levels previously analysed (item 2.2). Figure 5 shows the chromatogram of the extract obtained from the French press coffee beverage that used dark roasted coffee beans. Figure 6, on the other hand, gathers the information on C&K and their degradation product (C&K-Der.) contents in different coffee beverages, all derived from coffee beans subjected to light, medium and dark roasting.

Among the evaluated beverages, filtered coffee showed the lowest extraction efficiency of total diterpenes (C&K and its degradation derivatives) (Figure 6). This result is in agreement with the articles that describe the low concentration of C&K for this beverage due to the poor desorption of these compounds together with the coffee grounds, which are nothing more than the roasted and ground coffee beans that are retained in paper or cotton filters after preparing the beverage [29,30,38]. Among the other beverages, boiled coffee was the one that provided the highest extraction yield, followed by the French press, Moka and Turkish (Figure 6). 

This result is consistent with other authors, whose variation in levels between these beverages may occur due to the randomness of the particular components of each method of preparation employed (level of roasting and particle size of ground beans, proportion between coffee powder and water and temperature used during extraction) as well as the beans used (genetic load, maturation stage, degree of dehydration during the drying stage and action of microorganisms and/or pests) [8,28,38].

As for roasting levels, the highest C&K values were observed for beans submitted to light roast, since the increase in time and temperature, necessary for medium and dark roasting, promote more pyrolysis reactions and an increase in the respective roasting products degradation (C&K-Der). The result is a reduction in the content of C&K in the most intense roast level and an increase in the derivatives of C&K degradation (C&K-Der.), the sum of which exceeds 1 g L^−1^ in the boiled beverage (Figure 5B,C)—a result slightly higher than that found by Erny et al. [27]. 

A curious fact was that the total contents of diterpenes present in boiled coffee beverages (Figure 6: 1236, 1398 and 849 mg for light, medium and dark roasting) were higher than those observed for the respective beans used (Table 1: 1005, 1297 and 740 mg for light, medium and dark roasts). This is the result of the evaporation of water during the preparation of the beverage, which concentrates these diterpenes in them.

## 3. Materials and Methods

### 3.1. Standards and Reagents

Methanol and *tert*-butyl methyl ether (TBME) (HPLC/Spectro grade, Tedia, Brazil) were purchased from Tedia (Rio de Janeiro, Brazil). Potassium hydroxide (KOH) was purchased from Vetec (Duque de Caxias, Brazil). Cafestol and kahweol diterpenes were isolated from green Arabica coffee beans (Rio de Janeiro, Brazil) according to Novaes et al. [64].

### 3.2. Raw and Roasted Coffee Samples

A single lot containing 3 kg of green 100% Arabica coffee beans (Armazém do Café, Rio de Janeiro, Brazil) was purchased and divided into 100 g portions, which were roasted in three different conditions (light roast: 230 °C and 12 min, 85–95 Å; medium roast 240 °C and 14 min, 55 Å; and dark roast: 250 °C and 17 min, 35 Å) in a bench roaster (Coffee Bean Roaster, model CBR-101—Gene Café, Gyeonggi-Do, Korea).

The roasted beans were ground (IKA^®^ A11 basic grinder, Campinas, SP, Brazil) and screened (850 μm sieves, Bertel Industria Metalurgica LTDA, Caieiras, Brazil). From these samples, five types of coffee beverages were prepared: filtered, boiled, Moka, French press and Turkish coffee. The preparation of each of the coffee beverages was performed in a proportion of 100 g L^−1^ as recommended by the Brazilian Coffee Industry Association [65].

#### Preparation of Coffee Beverages

- Filtered coffee: The filtered coffee beverage was made in an electric coffee maker (model PH14, Philco) by pouring 150 mL of mineral water into its respective compartment, and 15 g of coffee powder was placed in a filter paper (model 102, 3 Corações, Nova Iguaçu). The coffee maker was turned on, heating the water to a temperature of less than 100 °C, which percolated through the coffee powder on the filter and was collected in a small jar below.
- Boiled Coffee: Boiled coffee was made by placing 15 g of coffee powder in 150 mL of mineral water and boiling for 10 min. Subsequently, the drink was left to rest for 5 min to decant the suspended solids and then collected for analysis.
- Moka (Italian coffee): The bottom compartment of the Moka coffee maker (Bialetti©, six-cup model), also known as an Italian coffee maker, was filled with 150 mL of mineral water, not exceeding the reference level. The coffee powder (15 g) was placed in the funnel without compacting it. The upper part was screwed onto the bottom part, and the coffee maker was placed under heating. When the coffee finished passing under the funnel filled with powder, the coffee maker was removed from the heating and the beverage collect for analysis.
- French Press: 15 g of powder was added to the French press coffee maker (Bialetti©, three-cup model), followed by 150 mL of hot mineral water (90 ± 2 °C). The plunger assembly was placed in the upper part, closing the coffee maker and leaving the drink to infuse for 5 min. After that, the plunger was pushed to the end to complete the extraction.
- Turkish Coffee: 15 g of coffee powder was poured into the *cezve*—a Turkish coffee pot (Ibrik©, 0.35 L model)—with mineral water (150 mL). The coffee maker was placed under heating. When boiling began and the foam went to the coffee maker’s top, it was removed from heating. The coffee maker heating and its retirement were repeated twice. The beverage was carefully poured into a cup for analysis without transferring the coffee grounds.

### 3.3. Extraction of Diterpenes in Green and Roasted Coffee Beans

The extraction of diterpenes from coffee beans was performed following the optimized procedure previously published by our group [64]. Ground green or roasted coffee beans (500 mg) were added to a 4 mL flask with a magnetic stir bar and 2 mL of 1 M KOH in methanol (56.1 g L^−1^). The flask was closed with an airtight screw cap and placed on a silicon carbide platform (Anton Paar GmbH, Graz, Austria), and this was placed on a heating plate equipped with a magnetic stirrer and temperature sensor (Gehaka, Real Parque, São Paulo, Brazil). Methanolysis reactions took place at 60 °C under vigorous stirring for 90 min. 

Subsequently, 2 mL of distilled water was added, followed by extraction of the methanolized lipid fraction with 2 mL of TBME, which was repeated two more times. Finally, the organic extracts were grouped and washed with 2 mL of Milli-Q water, concentrated in a rotary evaporator, resuspended in 1 mL of methanol and reserved for analysis by GC (item 5.5).

### 3.4. Extraction of Diterpenes in Coffee Beverages

The extraction of diterpenes in coffee beverages was performed according to the method optimized by Moeenfard et al. [28]. After preparing the coffee beverages, a 2.5 mL aliquot was collected and transferred to a 4 mL flask with a magnetic stir bar, and 3.0 g of potassium hydroxide was added for the hydrolysis reaction. The flask was closed and placed on a silicon carbide platform heated to 80 °C for 60 min under vigorous stirring, where the C&K esters were converted to the respective alcohols—free diterpenes—and fatty acid salts. 

Then, the diterpenes were extracted twice with 5 mL of ethyl ether (P.A. ACS, Vetec, Duque de Caxias, Brazil), the solution was centrifuged (3000 rpm for 5 min), and the aqueous phase was discarded. The organic phases were pooled and washed with 5 mL of 2 M sodium chloride solution (NaCl P.A. ACS, Vetec), centrifuged again, and then the organic phase collected. This was concentrated by rotary evaporation and resuspended with 500 μL of methanol, followed by GC analysis (item 5.5).

### 3.5. GC Analysis

An Agilent Technologies (Palo Alto, CA, USA) gas chromatograph, model 6890 1530N, equipped with an HP 7673 autosampler, a flame ionization detector (FID) for quantitative analysis and an Agilent 5973N quadripolar mass spectrometer (MS) was used for compound identification. Helium (99.9992%) was used as the carrier gas at a flow rate of 2 mL min^−1^ in constant flow mode. Split injector (1:50) heated to 330 °C in pulsed mode (25 psi during the initial 15 s). 

The injection volume was 1.0 μL. Capillary column DB-17HT (50% phenyl 50% methylsiloxane, 10 m × 0.25 mm × 0.15 μm) was used, and the oven of the equipment programmed at 90 °C (0.25 min) was heated at a rate from 12 °C min^−1^ to 320 °C. The FID or MS transfer lines were maintained at 340 °C. MS conditions include ion source temperature at 230 °C, quadrupole at 200 °C, acceleration voltage of 200 eV above the set with an ionization voltage of 70 eV. Mass spectra were obtained in SCAN mode in the *m/z* range 50–800 Da at a rate of 1.99 scans s^−1^.

Acceptance criterion for identification of compounds were based on NIST/EPA/NIH Mass spectral Library (Version 2.2, 2014) Match and Relative Match scores ≥ 750. Experimental retention index (*I_Exp_*) with *n*-alkanes standards was obtained.

## 4. Conclusions

The phytochemicals cafestol and kahweol (C&K) were quantified in raw and roasted Arabica coffee beans, which are partially degraded from light roasting, whose process is intensified in medium and dark roasts. About 16 compounds were identified as the pyrolysis products of C&K diterpenes determined from the peculiar fragmentation profile of C&K in a GC-MS. Ten were derived from kahweol and six from cafestol, among derivative aldehydes, ethers and dehydration products.

All these diterpene compounds were obtained from the raw and roasted beans by a reactive extraction with KOH in methanol, which favoured the yielding. The final isolation was performed by liquid–liquid extraction with TBME. The efficiency of the approached extraction steps indicated the rigidity of the solid matrix as a variable—greater for the raw grain than for the roasted one. The roasting favoured the lipid extractions by pressing or solid–liquid procedures. Light and medium roasting allow higher yields without considerable changes in the chemical composition of the extracts.

A total of 15 different hot coffee extraction procedures (filtered, boiled, mocha and French and Turkish press) were prepared from roasted coffee beans (light, medium and dark roast). The reactive KOH extraction of C&K diterpenes and their degradation products was performed. This was additionally improved by the saline effect discussed in the main text. All the diterpenes were found in the beans. We also compared the efficiency of the water extraction of these phytochemicals with different devices. By far, the boiled technique presented much higher extraction yield.

Although, in the literature, some of these compounds have been identified in roasted coffee beans, they have never been observed in coffee beverages. Roasting temperature and time are the main factors for producing these derivatives. Therefore, they need to be carefully investigated to understand the kinetics of the formation of each of these new molecules and their relationship with the final quality of the beverage (perceived by the consumer and by professional evaluators). Espresso, capsule espresso, vent and cold and ice coffee brew were not evaluated in this publication.

An unequivocal identification of these diterpenes is necessary, which must be done by structural elucidation techniques, such as HRMS, NMR, IR and others. Thus, the scale-up of the extraction procedure becomes an alternative for obtaining these molecules in sufficient quantities for their chemical characterization and application to tests that evaluate their pharmacological properties and toxicity. From then on, it will be possible to know what is ingested when consuming a cup of coffee, especially for dark and very dark roasted coffee beans, as these are have the highest content of these degradation products.

## Figures and Tables

**Table 1:** Diterpene content (mg 100 g^−1^) in green and roasted coffee beans.

Sample	Kahweol Derivatives	Cafestol Derivatives	Kahweol	Cafestol	TOTAL
Green bean	0.000	0.000	0.426	0.371	0.797
Light roast	0.007	0.011	0.510	0.477	1.005
Medium roast	0.052	0.096	0.581	0.568	1.297
Dark roast	0.173	0.265	0.143	0.159	0.740

## Supplementary Materials

The following supporting information can be downloaded at: https://www.mdpi.com/article/10.3390/plants12081580/s1, Table S1: Atomic masses of fragments and molecular ions of green and roasted coffee diterpenes. Black font corresponds to the ions observed in the mass spectra (Figure 4, main text), while those in gray color should exist but were not observed; Table S2: Effect of KOH content upon diterpene concentration.
