# Supplementary material for: Extraction of Diterpene-Phytochemicals in Raw and Roasted Coffee Beans and Beverage Preparations and Their Relationship
Source: Plants (Basel). 2023 Apr 7;12(8):1580. doi: 10.3390/plants12081580 (PMC10145731; doi:10.3390/plants12081580)
Supplement: Supplementary file 1 [file plants-12-01580-s001.zip › plants-2244307-supplementary.pdf]

Supplementary Materials – Plants

Special Issue "Extraction Technologies, Isolation, Separation, and Application of Phytochemicals"

Extraction of diterpene-phytochemicals in raw and roasted coffee beans and beverage preparations and their relationship

Fábio Junior Moreira Novaes[1,], Maria Alice Esteves da Silva[2], Diana Cardoso Silva[1], Francisco Radler de Aquino Neto[3], Claudia Moraes Rezende[2]*

[1] Universidade Federal de Viçosa, Departamento de Química, Avenida Peter Henry Rolfs, s/n, Campus Universitário, Viçosa, MG 36570-900, Brazil

[2] Universidade Federal do Rio de Janeiro, Instituto de Química, Laboratório de Análise de Aromas, Avenida Athos da Silveira Ramos, 149, Bloco A, Instituto de Química, Sala 626A, Rio de Janeiro, RJ 21941-895, Brazil

[3] Universidade Federal do Rio de Janeiro, Instituto de Química, Laboratório de Apoio ao Desenvolvimento Tecnológico (LADETEC), Avenida Horácio Macedo, 1281, Polo de Química, bloco C, Rio de Janeiro, RJ 21941-598, Brazil

*Corresponding author: Phone: +55 31 3612-6635.

E-mails: fabio.novaes@ufv.br (Fábio J. M. Novaes); esteves358@gmail.com (Maria A. E. Silva); diana.cardoso@ufv.br (Diana C. Silva); radler@iq.ufrj.br (Francisco R. Aquino Neto); crezende@iq.ufrj.br (Claudia M. Rezende).

Content:

Table S1. Atomic masses of fragments and molecular ions of green and roasted coffee diterpenes. Black font corresponds to the ions observed in the mass spectra (Fig. 4, main text), while those in gray color should exist but were not observed.

Table S2. Effect of KOH content upon diterpene concentration.

Table S1. Atomic masses of fragments and molecular ions of green and roasted coffee diterpenes. Black font corresponds to the ions observed in the mass spectra (Fig. 4, main text), while those in gray color should exist but were not observed.

N°	t_R	Name	Fragments of diterpene B-ring			$[M]^+$	M-18	M-31	M-18 -31	M-18 -31 -15	M-31 -28	M-15	M-15 -18	M-15 -28	M-function	M-15 -18 -28	M-15 -18 -28 -14
1	11.025	Cafestol derivative (#1)	133	147	161	282	-	-	-	-	-	267	-	239	-	-	-
2	11.347	Cafestol derivative (#4)	133	147	161	282	-	-	-	-	-	267	-	239	-	-	-
3	12.486	Kahweal (#8)	131	145	158	296	-	-	-	-	-	281	-	-	267	-	-
4	12.553	Dehydro-kahweol derivative (#9)	131	145	158	296	278	265	247	232	237	281	263	253	-	235	221
5	12.697	Dehydro-kahweol derivate (#11)	131	145	158	296	278	265	247	232	237	281	263	253	-	235	221
6	12.757	15,16-Dehydro-kahweol (#12)	131	145	158	296	278	265	247	232	237	281	263	253	-	235	221
7	12.855	Dehydro-kahweol derivate (#13)	**131**	**145**	158	**296**	278	**265**	247	232	237	281	263	253	-	235	221
8	12.898	Cafestal (#14)	**133**	**147**	**161**	**298**	-	-	-	-	-	**283**	-	-	**269**		
9	12.964	15,16-Dehydro-cafestol (#15)	**133**	**147**	**161**	**298**	280	267	247	232	239	**283**	**265**	255	-	237	223
10	13.048	Dehydro-kahweol derivate (#17)	**131**	**145**	**158**	**296**	278	265	247	232	237	281	263	253	-	235	221
11	13.065	Dehydro-kahweol derivate (#19)	**131**	**145**	158	298	280	267	249	234	239	283	265	255	-	237	223
12	13.122	Dehydro-kahweol derivate (#19)	131	145	158	296	278	265	247	232	237	281	263	253	-	235	221
13	13.170	Cafestol derivative (#20)	**133**	**147**	161	300	282	269	251	236	241	285	267	257	-	239	225
14	13.206	16-*O*-isobutyl-kahweol (#21)	131	145	158	370	-	339	-	-	311	355	337	327	296	309	295
15	13.382	16-*O*-isobutyl-cafestol (#22)	133	147	161	372	-	341	-	-	313	357	339	329	298	311	297
16	13.915	Kahweol (#24)	**131**	**145**	158	**314**	**296**	**283**	265	250	255	299	281	271	-	253	239
17	14.107	Cafestol (#25)	**133**	**147**	**161**	**316**	**298**	**285**	**267**	**252**	257	301	283	273	-	255	241
18	14.239	*Seco*-kahweol (#26)	**131**	**145**	158	**314**	**296**	**283**	265	250	255	299	281	271	-	253	239

[M]+: molecular ion; M-X correspond to fragments from specific losses: 18 (H_2O), 31 ($-CH_2-OH$), 15 ($-CH_3$), 28 (=CO or $-CH_2=CH_2$), function (aldehyde = -CHO = 29 or ether group), 14 ($=CH_2$).

Table S2. Effect of KOH content upon diterpene concentration.

KOH (g)	[C&K, mg L^{-1}]	Normalized Value
3,0	152.8	100.0
1,0	140.2	91.8
0,3	122.0	79.9
